# Characteristics of clinical trials related to hip fractures and factors associated with completion

**DOI:** 10.1186/s12891-022-05714-x

**Published:** 2022-08-16

**Authors:** Shengjie Wang, Fan Xiong, Yanzheng Gao, Mingxing Lei, Xianlong Zhang

**Affiliations:** 1 Department of Orthopaedic Surgery, Shanghai Sixth People’s Hospital Affiliated to Shanghai Jiao Tong University, Yishan Road 600#, Shanghai, 200233 China; 2 Department of Orthopedic Surgery, People’s Hospital of Macheng City, Huang Gang, 438399 China; 3 Department of Orthopaedic Surgery, Henan Provincial People’s Hospital, Zhengzhou, 450003 China; 4 Chinese PLA Medical School, 28 Fuxing Road, Beijing, 100853 China; 5 Department of Orthopedic Surgery, Hainan Hospital of PLA General Hospital, Sanya, China; 6 The National Clinical Research Center for Orthopedics, Sports Medicine & Rehabilitation, PLA General Hospital, Beijing, China

**Keywords:** Clinical trials, Hip fractures, ClinicalTrials.gov, Completeness, Drugs/biologics

## Abstract

**Background:**

This study aimed at investigating the characteristics of clinical trials related to hip fractures that were registered at ClinicalTrials.gov. It also aimed to identify potential risk factors associated with completion.

**Main body:**

We obtained 733 clinical studies related to hip fractures from the ClinicalTrials.gov database and included 470 studies in the analysis. These clinical trials were divided into behavioral, drug/biological, device, procedure, and other categories based on intervention types. Clinical trials investigating drugs or biologics were categorized based on the specific agents administered in each trial. Multiple logistic and Cox regression models were used to test the ability of 24 potential risk factors in predicting recruitment status and completion time, respectively. Among the included clinical trials, 44.89% (211/470) had complete recruitment status. The overall median completion time was 931.00 days (95% confidence interval [CI]: 822.56–1039.44 days). The results of only 8.94% (42/470) of clinical trials were presented on the ClinicalTrials.gov website. Bupivacaine (a local anesthetic) was most commonly investigated (in 25 clinical trials); this was followed by ropivacaine (another local anesthetic, 23 clinical trials) and tranexamic acid (a hemostatic, 21 clinical trials). Multivariate analysis showed that trials including children (*P* = 0.03) and having National Institutes of Health funds (*P* < 0.01) had significantly higher rates of complete recruitment. Higher enrollment (*P* < 0.01), National Institutes of Health funding (*P* < 0.01), location in the United States (*P* = 0.04), and location in Europe (*P* = 0.03) predisposed to longer completion time, while studies involving drugs/biologics (*P* < 0.01) had shorter completion times.

**Conclusions:**

A considerable proportion of clinical trials related to hip fractures were completed, but the results of only a small fraction were presented on the ClinicalTrials.gov website. The commonly investigated drugs focused on anesthesia, pain relief, and hemostasis. Several independent risk factors that affect recruitment status and completion time were identified. These factors may guide the design of clinical trials relating to hip fractures.

**Supplementary Information:**

The online version contains supplementary material available at 10.1186/s12891-022-05714-x.

## Background

Hip fractures have become a significant global public health problem due to the gradual ageing of the population. Elderly patients, especially women, usually suffer from a reduction of bone mineral density and an increasing risk of falls, both of which are responsible for the pathogenesis of hip fractures. According to the literature, this disease approximately affects 18% females and 6% males globally. It has been estimated that the number of hip fractures worldwide will rise from 1.26 million (30 years previously) to approximately 4.5 million in 2050 [1]. Owing to long-term hospitalization and rehabilitation, the corresponding economic impact on healthcare is expected to be enormous. In addition, hip fractures may subsequently lead to disabilities, mortality, secondary fractures, and cardiovascular diseases [2], among other conditions.

A series of clinical trials have been conducted with the aim of preventing or treating hip fractures. These clinical trials are mostly registered on the ClinicalTrial.gov database, which was launched in 2000; it is the largest global trial repository that allows patients, medical workers, and the public to easily access basic information about publicly and privately supported clinical research on a variety of diseases or conditions. The International Committee of Medical Journal Editors has declared that only clinical studies registered at ClinicalTrials.gov before patient recruitment may be considered for publication. The registration of clinical trials became a prerequisite for publication after the issue of the Food and Drug Administration Modernization Act of 1997 [3]; in this context, there are legal repercussions if the new registrations are not accurate [4].

Studies pertaining to orthopedic trauma trials published ten years previously [3], prospective spine studies [5], spinal cord injury trials [6], rheumatoid arthritis trials [7], and sports medicine-related randomized controlled trials [8] have been published following registration in Clinicaltrials.gov. However, these studies aimed at investigating the relationship between clinical trials and corresponding publications. Articles summarizing the characteristics of clinical trials relating to orthopedics, and particularly hip fractures, are scarce. A good understanding of the characteristics may be able to guide future research on hip fractures. In addition, the identification of potential risk factors associated with completion may help to reduce wastage of resources and improve efficiency of clinical trials specifically related to hip fractures.

Therefore, this study aimed to investigate the characteristics of clinical trials related to hip fractures and identify potential risk factors associated with completion, as this could reduce wastage of resources and improve efficiency. We hypothesized that this study could provide valuable insights into the characteristics associated with recruitment status and completion time in clinical trials related to hip fractures.

## Methods

### Registry search

We searched the ClinicalTrials.gov web site for clinical studies related to hip fractures (https://clinicaltrials.gov/, accessed on 10 July 2021). The search was conducted using the following keywords in the “condition or disease” field in ClinicalTrials.gov: “hip fractures” OR “trochanteric fractures” OR “intertrochanteric fractures” OR “subtrochanteric fractures” OR “femoral neck fractures” OR “femur neck fractures” OR “femoral head fractures” OR “femur head fractures.” We then downloaded all results and reviewed each trial identified by the search. Studies were excluded if they did not meet the requirement of clinical trials adopted by the World Health Organization. As per the requirements, a clinical trial involves prospective assignment of human participants to one or more groups and treatment with medical interventions for evaluating the effects on health-related outcomes. According to the definition, observational studies were excluded. Studies that were withdrawn, not related to hip fractures, and having missing data were also excluded.

Patients were not involved in any aspect of the study design or conduct, or in the development of the research question. The present study analyzed existing publicly available data; active patient recruitment was therefore not performed for data collection. The Ethics Committee Board of the Shanghai Sixth People’s Hospital Affiliated to Shanghai Jiao Tong University waived the need for informed consent, as all data were publicly available at https://clinicaltrials.gov/. This study adhered to the principles of the Declaration of Helsinki.

### Classification of clinical trials

After exclusion, we divided the remaining clinical trials into behavioral, drug/biological, device, procedure, and other intervention categories based on intervention type. Clinical trials investigating drugs or biologics were categorized based on the agents administered in each trial. We have discussed the agents tested in three or more clinical trials and the function of these agents. If a particular drug or biologic was administered to participants in any of the therapeutic arms of the trial, this drug or biologic was regarded as being involved in the clinical trial.

A bar chart was drawn to identify the trends of newly registered clinical trial numbers with time; the trial location categories were as follows: China, the United States, Europe, Canada, and others. A bar chart was also constructed on the relative proportion of newly registered clinical trials on ClinicalTrials.gov, based on the five different locations.

### Characteristics of clinical trials

We have presented data pertaining to the recruitment status, whether study results were shown on the ClinicalTrials.gov website, start date (years), outcome measures including mortality, participant’s gender and age, phases, number of enrollments, funders, study design, primary purpose, interventions, trial locations, and completion time.

Recruitment status included the following categories: not yet recruiting, recruiting, enrolling by invitation, active, not recruiting, suspended, completed, and unknown. If the outcome measures of a trial involved mortality, the trial was considered in the “outcome measures including mortality” category. During analysis of the data, clinical trials involving phases 1 and 2 were regarded as phase 2 trials, while those marked as phase 2 and 3 were considered as phase 3. Funders included the National Institutes of Health (NIH), industry, and others. Study designs included the following factors: allocation, intervention model, and masking. The primary purpose included the following categories: diagnostic, health service research, prevention, supportive care, treatment, and others. Interventions included behavioral, drug/biological, device, procedure, and others. Trial locations were categorized as follows: China, the United States, Europe, Canada, and others. Completion time was defined as the time interval between the start and completion dates. The completion date was divided into actual and estimated dates. The actual completion date of a clinical study was defined as that on which the last participant was examined or received an intervention for collection of final data for the primary outcome measure; the estimated completion date was the expected primary completion date for the study.

### Potential risk factors associated with recruitment status, completion time, and successful trial completion on ClinicalTrials.gov

We identified 24 potential risk factors for predicting recruitment status, completion time, and successful trial completion; these included outcome measures including mortality (yes vs. no), gender (male vs. female vs. both), participants including children (yes vs. no), age including older adults (yes vs. no), phases (not applicable, phase 1, phase 2, phase 3, or phase 4), enrollment (≤ 50 vs. > 50, ≤ 100 vs. > 100, ≤ 200 vs. > 200, and ≤ 400 vs. > 400), being funded by the NIH (yes vs. no), being funded by industry (yes vs. no), allocation (not applicable, non-randomized, or randomized), intervention model (single, sequential, parallel, factorial, or crossover), masking (none, single, double, triple, or quadruple), primary purpose (diagnostic [yes vs. no], health service research [yes vs. no], prevention [yes vs. no], supportive care [yes vs. no], and treatment [yes vs. no]), interventions (behavioral [yes vs. no], drug/biological [yes vs. no], device [yes vs. no], and procedure [yes vs. no]), and locations (in China [yes vs. no], the United States [yes vs. no], Europe [yes vs. no], and Canada [yes vs. no]).

Participants aged 17 years or less were defined as children; those older than 65 years were defined as older adults. The industry funders included pharmaceutical and device companies. Allocation referred to the method used to assign participants to a therapeutic arm; this included the following categories: not applicable, non-randomized, and randomized. The intervention model referred to the general design of the strategy for assigning interventions to participants. Masking indicated the clinical trial design strategy; it included open label, single blind masking, and double-blind masking approaches, where one or more parties were unaware of the specific participants who were assigned to a particular intervention. Successful trial completion was defined as the availability of results on the ClinicalTrials.gov website.

### Statistical analysis

We have presented descriptive data for each category, including proportions for categorical data and medians with 95% confidence intervals (CIs) for completion time. Univariate and multivariate analyses of characteristics for recruitment status and final successful trial completion (having results on the ClinicalTrials.gov website) were both performed using simple and multiple logistic regression models. In the models, recruitment status was classified into two or seven categories. Among the significant variables identified on multivariate analysis, the distribution of recruitment status was further investigated using the Chi-square or Mantel–Haenszel Chi-square tests. Availability of results on the ClinicalTrials.gov website was classified into two categories (yes vs. no). The predictive performance of significant variables for successful trial completion was measured by area under the receiver operating characteristic (AUROC) curves. Simple and multiple Cox regression models were used to test the ability of potential risk factors in predicting completion time. Cumulative completion curves obtained using the log-rank test have been presented. Subgroup analysis was performed to test the ability of potential risk factors in predicting actual completion time; a *P* value of 0.05 or less (two-sided tests) was considered as statistically significant. All data processing, statistical analysis, and plotting were performed using SAS 9.4, IBM SPSS Statistics 21.0, and R 4.0.5 (https://www.r-project.org/) software.

## Results

### Characteristics of clinical trials related to hip fractures

**Fig. 1:**
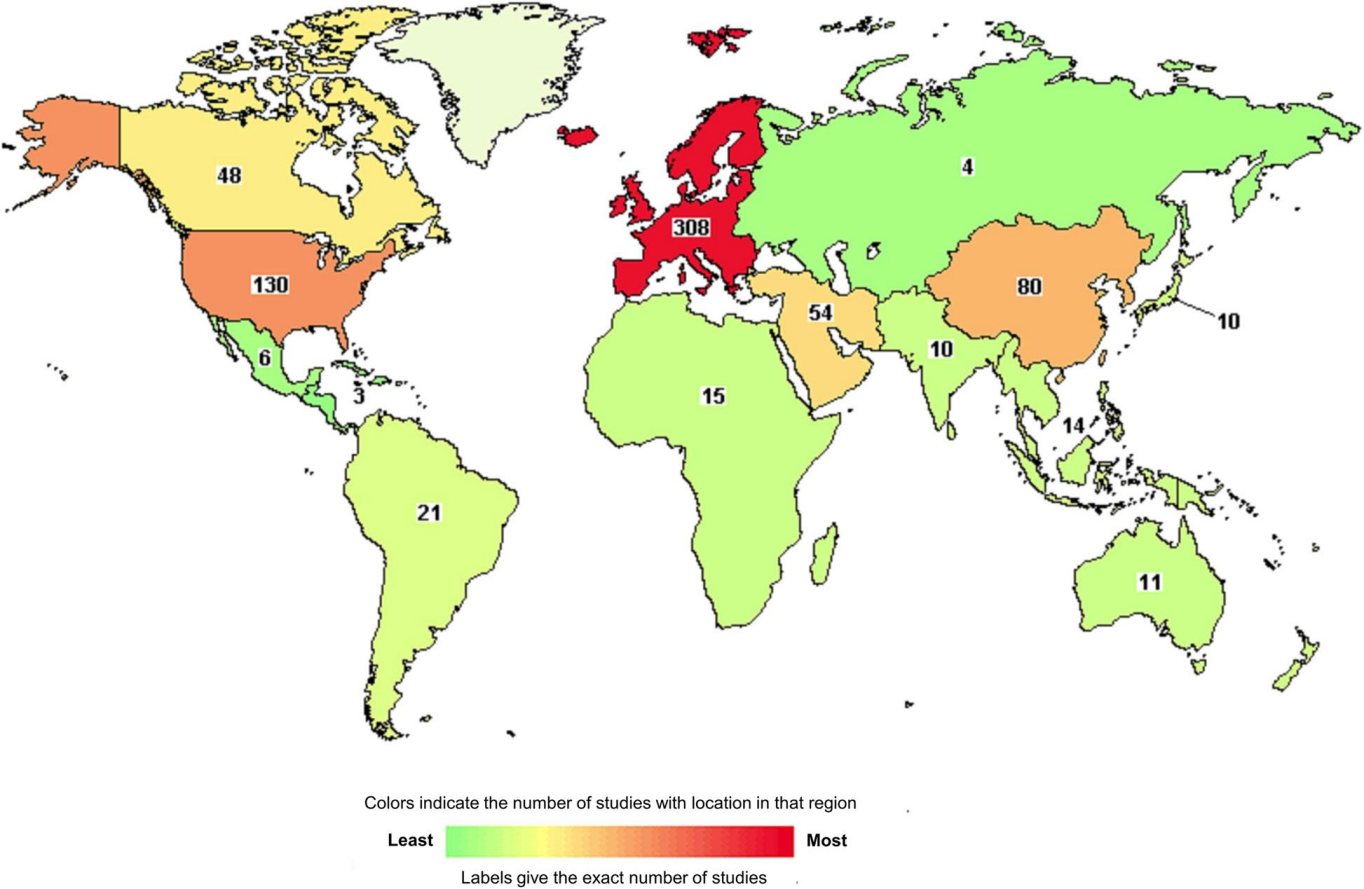
Heat map of the number of clinical studies related to hip fracture worldwide (Green indicates the least; Red indicates the most)

**Fig. 2:**
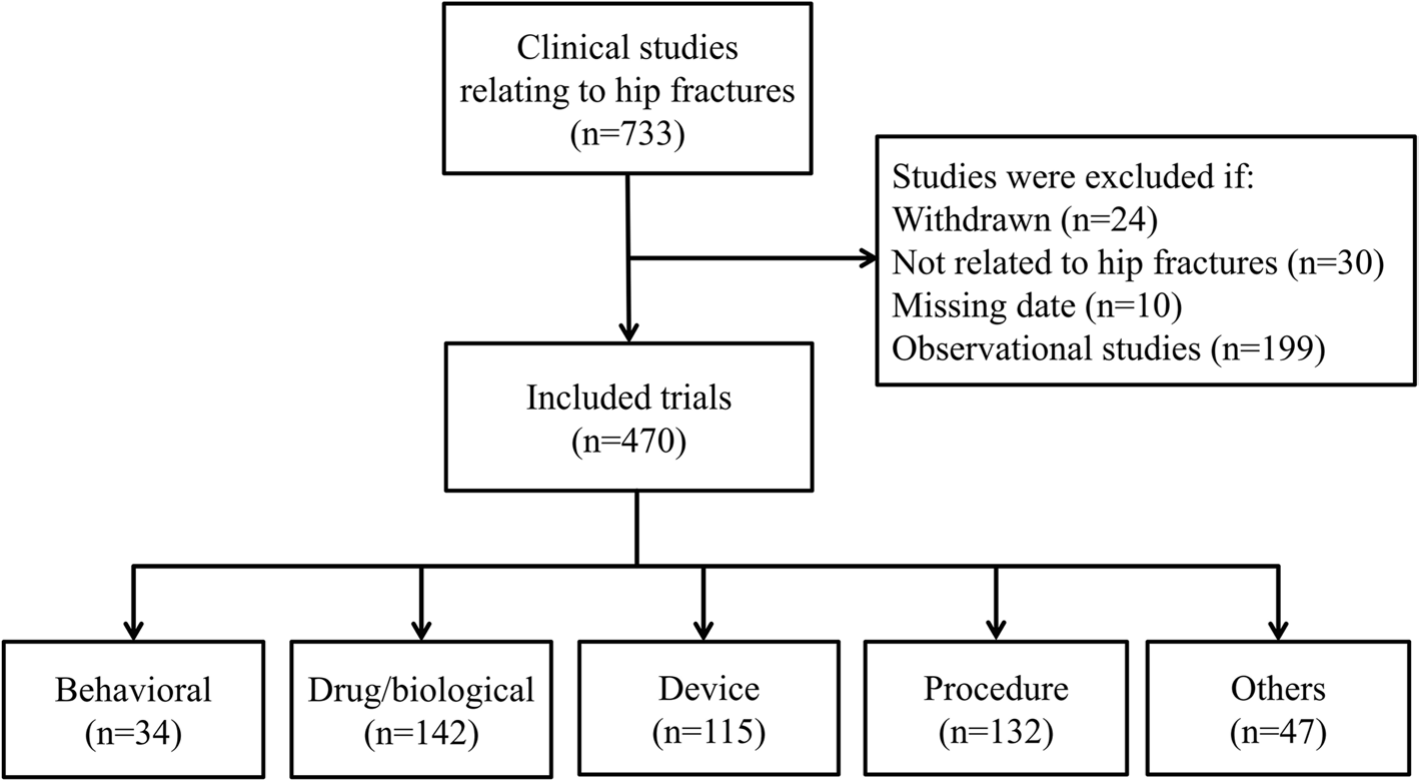
Flow chart of included clinical trials related to hip fracture registered at ClinicalTrials.gov

A total of 733 clinical studies related to hip fractures were obtained from the ClinicalTrials.gov database. A map of the worldwide distribution and number of clinical studies is shown in Fig. 1. It indicates that a multitude of clinical studies related to hip fractures were performed at Europe, followed by the United States. Based on the exclusion and inclusion criteria, 470 clinical trials were finally included in the analysis. Figure 2 shows the flow chart of the included clinical trials.

Clinical trials that had complete recruitment status accounted for 44.89% (211/470) of all included studies (Table 1). The overall median completion time was 931.00 (95% CI: 822.56–1039.44) days. Only the results of 8.94% (42/470) of clinical trials were presented on the ClinicalTrials.gov website. Almost 98.09% (461/470) of the trials included older adults, as hip fractures usually tend to occur in the geriatric population due to osteoporosis. Regarding the phases, 67.02% (315/470) clinical trials were of the not applicable category, followed by phase 4 (15.96%, 75/470) and phase 3 (10.43%, 49/470) trials. Among the included clinical trials, 75.74% (356/470) had 200 or fewer participants, only 3.83% (18/470) were funded by the NIH, and 39.57% (186/470) did not employ masking; all these may indicate that the majority of clinical trials were relatively small. The most common primary purpose was treatment (68.09%, 320/470) and the most common intervention was drug/biological therapy (30.21%, 142/470).

**Table 1:** Characteristics of clinical trials relating to hip fractures

Characteristics	Samples (*n* = 470)
Recruitment status	
Completed	44.89% (211/470)
Others	55.11% (259/470)
Recruitment status	
Not yet recruiting	6.81% (32/470)
Recruiting	15.11% (71/470)
Enrolling by invitation	1.49% (7/470)
Active, not recruiting	3.83% (18/470)
Suspended	9.57% (45/470)
Completed	44.89% (211/470)
Unknown	18.30% (86/470)
Study results	
No Results Available	91.06% (428/470)
Has Results	8.94% (42/470)
Start date (years)	
≦2010 years	29.36% (138/470)
> 2010 years and ≦2016 years	35.11% (165/470)
> 2016 years	35.53% (167/470)
Outcome measures including mortality	
Yes	17.02% (80/470)
No	82.98% (390/470)
Gender	
Male	0.85% (4/470)
Female	3.19% (15/470)
Both	95.96% (451/470)
Age including children	
Yes	8.51% (40/470)
No	91.49% (430/470)
Age including older adults	
Yes	98.09% (461/470)
No	1.91% (9/470)
Phases	
Not applicable	67.02% (315/470)
Phase 1	0.85% (4/470)
Phase 2	5.74% (27/470)
Phase 3	10.43% (49/470)
Phase 4	15.96% (75/470)
Enrollment	
≤ 50	26.38% (124/470)
> 50 and ≤ 100	26.81% (126/470)
> 100 and ≤ 200	22.55% (106/470)
> 200 and ≤ 400	14.47% (68/470)
> 400	9.79% (46/470)
Funded by the NIH	
Yes	3.83% (18/470)
No	96.17% (452/470)
Funded by industry	
Yes	15.32% (72/470)
No	84.68% (398/470)
Allocation	
Not applicable	11.06% (52/470)
Non-randomized	7.66% (36/470)
Randomized	81.28% (382/470)
Intervention model	
Single	16.60% (78/470)
Sequential	0.64% (3/470)
Parallel	77.23% (363/470)
Factorial	1.91% (9/470)
Crossover	3.62% (17/470)
Masking	
None	39.57% (186/470)
Single	26.60% (125/470)
Double	17.66% (83/470)
Triple	5.96% (28/470)
Quadruple	10.21% (48/470)
Primary purpose	
Diagnostic	3.40% (16/470)
Health service research	2.77% (13/470)
Prevention	15.74% (74/470)
Supportive care	5.74% (27/470)
Treatment	68.09% (320/470)
others	4.26% (20/470)
Interventions	
Behavioral	7.23% (34/470)
Drug/biological	30.21% (142/470)
Device	24.47% (115/470)
Procedure	28.09% (132/470)
Others	10.00% (47/470)
Locations	
China	9.36% (44/470)
United States	21.28% (100/470)
Europe	41.70% (196/470)
Canada	6.60% (31/470)
Others	
Completion time (median, 95% CI, days)	931.00 (822.56–1039.44)

*NIH* National Institutes of Health, *CI* Confidence intervals

**Fig. 3:**
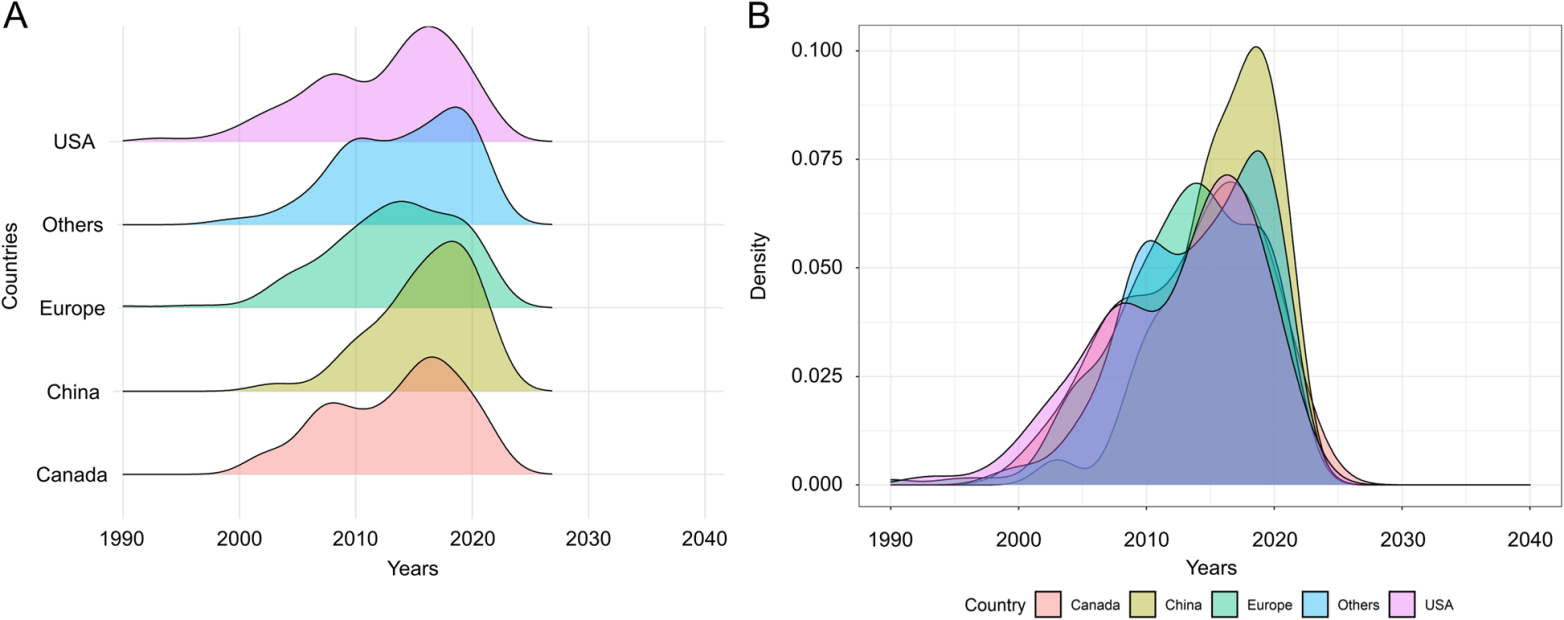
Density curve for clinical trials related to hip fracture registered at ClinicalTrials.gov. **a** Density curve with years plotted against the five areas (the United States of America [USA], Europe [Europ], China, Canada, and others); **b** Density curve with years plotted against the proportions in each area

After exclusion of irrelevant clinical studies, Europe continued to have the largest number of trials with a proportion of 41.70% (196/470); this was followed by the United States (21.28%, 100/470) and China (9.36%, 44/470). In general, based on the findings on the density curve (Fig. 3A), clinical trials on hip fractures were developed early in the United States since its curve began to steadily increase at the beginning of 1990, and the time was earlier than that of any other countries; in China, the number of clinical trials related to hip fractures mostly increased in the recent decades because its peak was much higher than that of any other countries during the recent decades (Fig. 3B).

Figure S1 shows trends of the number of newly registered clinical trials over time (years); it indicates that the number of newly registered clinical trials rose from below 10 at 20 years previously to approximately 70 in recent years. Figure S2 shows the relative proportion of new clinical trials registered on the ClinicalTrials.gov database, according to the five different locations.

### Clinical trials investigating the effects of drugs or biologics

We evaluated the specific agents tested in 3 or more clinical trials; 14 agents were finally included. Bupivacaine, a local anesthetic, was tested in up to 25 clinical trials related to hip fractures; this was followed ropivacaine, another local anesthetic which was investigated in 23 clinical trials, and tranexamic acid, a hemostatic which was tested in 21 clinical trials. More details are shown in Table 2. Regarding the function of the 14 agents, 4 were anti-osteoporotic, 3 were local anesthetics, and 2 were pain killers. Notably, the majority of local anesthetics used in the trials aimed at alleviating pain.

**Table 2:** Drugs or biologics investigated in clinical trials related to hip fractures

Drugs	Classifications	Number of clinical trials^a^
Bupivacaine	Local anesthetic	25
Ropivacaine	Local anesthetic	23
Tranexamic acid	Hemostatic	21
Fentanyl	Pain killer	9
Vitamin D	Anti-osteoporotic	8
Morphine	Pain killer	7
Teriparatide	Anti-osteoporotic	6
Dexmedetomidine	α2-adrenergic receptor agonist	4
Risedronate	Anti-osteoporotic	4
Lidocaine	Local anesthetic	4
enoxaparin	Antithrombotic agent	4
Sevoflurane	Inhalation anesthetic	4
Propofol	General anesthetic	4
Testosterone	Anti-osteoporotic	3

^a^Drugs investigated in three or more clinical trials are included in the table

### Risk factors associated with recruitment status

On categorizing the recruitment status into completed vs. others categories, univariate analysis showed that NIH funding (*P* < 0.01), interventions involving behavioral aspects (*P* = 0.04), and locations other than China (*P* = 0.02) were significantly associated with a higher rate of successfully completed recruitment (Table 3). In addition, clinical trials of a later phase tended to have a lower complete recruitment rate; however, this did not reach statistical significance (*P* = 0.07). On multivariate analysis, trials including children as participants (*P* = 0.03) and NIH funding (*P* < 0.01) had significantly higher rates of complete recruitment. In particular, clinical trials involving children were twice as more likely to complete recruitment than those not involving children. Clinical trials funded by the NIH were 8.71 times more likely to complete recruitment than those not funded by the NIH.

**Table 3:** Univariate and multivariate analyses of characteristics for completion of recruitment in clinical trials related to hip fractures

Characteristics	Completion rates	Simple logistic regression		Multiple logistic regression	
		**OR (95% CI)**	**P**	**OR (95% CI)**	**P**
Outcome measures including mortality					
Yes	45.00% (36/80)	1.01 (0.62–1.63)	0.98	0.97 (0.56–1.68)	0.91
No	44.87% (175/390)				
Gender					
Male	75.00% (3/4)	0.99 (0.40–2.45)	0.98	1.48 (0.51–4.32)	0.47
Female	53.33% (8/15)				
Both	44.35% (200/451)				
Age including children					
Yes	57.50% (23/40)	1.74 (0.90–3.35)	0.10	2.27 (1.11–4.68)	0.03
No	43.72% (188/430)				
Age including older adults					
Yes	45.34% (209/461)	2.90 (0.60–14.12)	0.19	3.45 (0.62–19.26)	0.16
No	22.22% (2/9)				
Phases					
Not applicable	40.63% (128/315)	1.11 (0.99–1.24)	0.07	1.10 (0.94–1.28)	0.22
Phase 1	50.00% (2/4)				
Phase 2	70.37% (19/27)				
Phase 3	61.22% (30/49)				
Phase 4	42.67% (32/75)				
Enrollment					
≤ 50	43.55% (54/124)	1.02 (0.88–1.17)	0.82	0.98 (0.82–1.15)	0.76
> 50 and ≤ 100	42.86% (54/126)				
> 100 and ≤ 200	47.17% (50/106)				
> 200 and ≤ 400	54.41% (37/68)				
> 400	34.78% (16/46)				
Funded by the NIH					
Yes	83.33% (15/18)	6.53 (1.86–22.87)	< 0.01	8.71 (2.13–35.61)	< 0.01
No	43.36% (196/452)				
Funded by industry					
Yes	50.00% (36/72)	1.27 (0.77–2.11)	0.34	1.61 (0.92–2.84)	0.10
No	43.97% (175/398)				
Allocation					
Not applicable	32.69% (17/52)	1.25 (0.94–1.66)	0.13	1.28 (0.80–2.05)	0.31
Non-randomized	50.00% (18/36)				
Randomized	46.07% (176/382)				
Intervention model					
Single	42.31% (33/78)	1.09 (0.89–1.35)	0.39	1.07 (0.78–1.49)	0.67
Sequential	0.00% (0/3)				
Parallel	45.18% (164/363)				
Factorial	66.67% (6/9)				
Crossover	47.06% (8/17)				
Masking					
None	44.09% (82/186)	1.01 (0.88–1.17)	0.86	0.86 (0.71–1.03)	0.10
Single	45.60% (57/125)				
Double	45.78% (38/83)				
Triple	42.86% (12/28)				
Quadruple	45.83% (22/48)				
Primary purpose					
Diagnostic					
Yes	25.00% (4/16)	0.40 (0.13–1.25)	0.12	0.72 (0.16–3.23)	0.67
No	45.59% (207/454)				
Health service research					
Yes	46.15% (6/13)	1.05 (0.35–3.18)	0.93	1.17 (0.27–5.07)	0.83
No	44.86% (205/457)				
Prevention					
Yes	40.54% (30/74)	0.81 (0.49–1.34)	0.41	0.80 (0.27–2.38)	0.69
No	45.71% (181/396)				
Supportive care					
Yes	44.44% (12/27)	0.98 (0.45–2.14)	0.96	0.90 (0.26–3.13)	0.87
No	44.92% (199/443)				
Treatment					
Yes	47.19% (151/320)	1.34 (0.90–1.99)	0.14	1.22 (0.46–3.27)	0.69
No	40.00% (60/150)				
Interventions					
Behavioral					
Yes	61.76% (21/34)	2.09 (1.02–4.28)	0.04	1.56 (0.67–3.64)	0.31
No	43.58% (190/436)				
Drug/biological agent					
Yes	50.00% (71/142)	1.34 (0.91–1.99)	0.14	1.11 (0.60–2.05)	0.75
No	42.68% (140/328)				
Device					
Yes	38.26% (44/115)	0.70 (0.45–1.07)	0.10	0.65 (0.38–1.13)	0.13
No	47.04% (167/355)				
Procedure					
Yes	40.91% (54/132)	0.80 (0.53–1.20)	0.28	0.86 (0.52–1.40)	0.54
No	46.45% (157/338)				
Location in China					
Yes	27.27% (12/44)	0.43 (0.22–0.85)	0.02	0.51 (0.23–1.15)	0.10
No	46.71% (199/426)				
Location in the United States					
Yes	49.00% (49/100)	1.23 (0.79–1.92)	0.35	0.82 (0.43–1.54)	0.53
No	43.78% (162/370)				
Location in Europe					
Yes	47.45% (93/196)	1.19 (0.83–1.73)	0.35	1.09 (0.66–1.82)	0.73
No	43.07% (118/274)				
Location in Canada					
Yes	45.16% (14/31)	1.01 (0.49–2.10)	0.98	0.97 (0.41–2.28)	0.94
No	44.87% (197/439)				

*NIH* National Institutes of Health, *OR* Odds ratio, *CI* Confidence intervals

On categorizing the recruitment status into seven subtypes, multivariate analysis showed that the outcome measures including mortality (*P* = 0.04), phase (*P* = 0.01), enrollment (*P* = 0.02), allocation (*P* = 0.05), diagnostic studies (*P* = 0.04), and location in the United States (*P* < 0.01) were significantly associated with recruitment status (Table S1). The distribution of recruitment status among outcome measures including mortality (*P* = 0.02), phases (*P* = 0.01), enrollment (*P* < 0.01), and location in the United States (*P* < 0.01) was also significantly different. More details are shown in Table S2.

### Risk factors associated with completion time

**Fig. 4:**
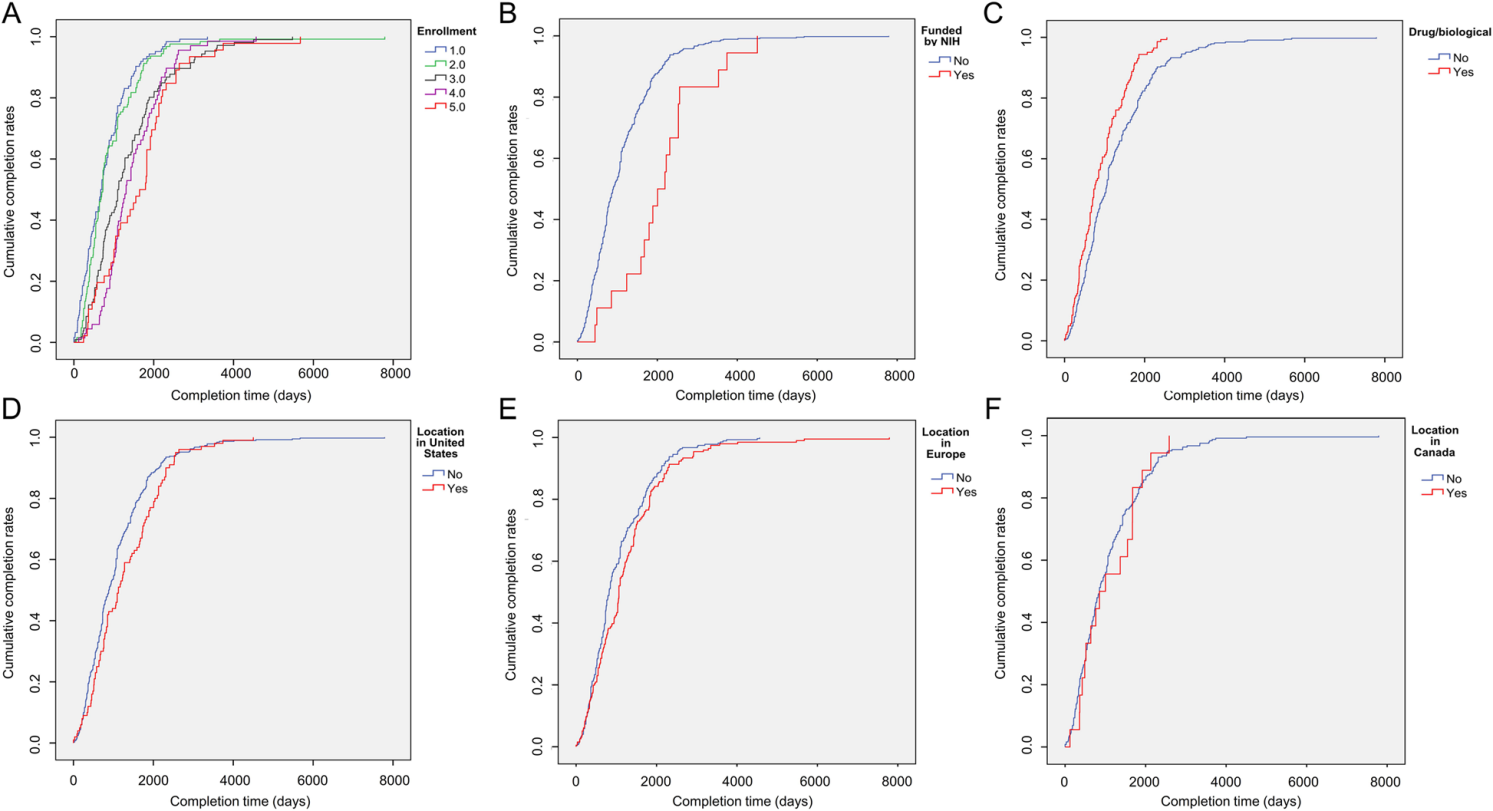
Cumulative completion curves. **a** Enrollment (1 indicates ≤ 50; 2 indicates > 50 and ≤ 100; 3 indicates > 100 and ≤ 200; 4 indicates > 200 and ≤ 400; and 5 indicates > 400; *P* < 0.01, log-rank test); **b** Funded by the National Institutes of Health (*P* < 0.01, log-rank test), **c** Interventions involving drugs/biologics (*P* < 0.01, log-rank test); **d** location in the United States (*P* = 0.04, log-rank test); **e** location in Europe (*P* = 0.03, log-rank test); f location in Canada (*P* = 0.95, log-rank test)

On multiple Cox regression, enrollment (hazard ratio [HR] = 0.77, 95% CI: 0.70–0.84, *P* < 0.01), being funded by the NIH (HR = 0.47, 95% CI: 0.28–0.82, *P* = 0.01), studies testing drugs/biologics (HR = 1.48, 95% CI: 1.07–2.05, *P* = 0.02), location in the United States (HR = 0.63, 95% CI: 0.46–0.87, *P* < 0.01), and location in Europe (HR = 0.62, 95% CI: 0.48–0.81, *P* < 0.01) were significantly relevant to completion time (Table 4). Higher enrollment (*P* < 0.01, log-rank test, Fig. 4A), being funded by the NIH (*P* < 0.01, log-rank test, Fig. 4B), location in the United States (*P* = 0.04, log-rank test, Fig. 4D), and location in Europe (*P* = 0.03, log-rank test, Fig. 4E) tended to prolong completion time, while studies involving drugs/biologics (*P* < 0.01, log-rank test, Fig. 4C) were associated with a shorter completion time. Similar results were obtained when subgroup analysis was performed after excluding studies having an estimated completion date. Enrollment (*P* < 0.01), being funded by the NIH (*P* = 0.01), location in the United States (*P* = 0.02), and location in Europe (*P* < 0.01) retained significance; however, studies involving drugs/biologics (*P* = 0.07) lost significance and location in Canada (*P* = 0.04) became significant. However, the log-rank test showed that location in Canada did not have significance (*P* = 0.95, log-rank test, Fig. 4F).

**Table 4:** Univariate and multivariate analyses of characteristics for completion time in clinical trials related to hip fractures

**Characteristics**	**Completion time (median, days)**	**Simple Cox regression**		**Multiple Cox regression**		**Multiple Cox regression**^a^	
		**HR (95% CI)**	**P**	**HR (95% CI)**	**P**	**HR (95% CI)**	**P**
Outcome measures including mortality							
Yes	1430.00	0.66 (0.52–0.84)	< 0.01	0.89 (0.67–1.17)	0.40	0.83 (0.57–1.22)	0.34
No	823.00						
Gender							
Male	122.00	1.42 (0.89–2.26)	0.13	1.05 (0.67–1.66)	0.83	0.87 (0.47–1.64)	0.67
Female	1065.00						
Both	931.00						
Age including children							
Yes	882.00	0.97 (0.70–1.34)	0.84	1.00 (0.70–1.43)	1.00	0.83 (0.52–1.34)	0.45
No	945.00						
Age including older adults							
Yes	931.00	1.00 (0.51–1.93)	0.99	1.56 (0.74–3.27)	0.24	0.87 (0.24–3.18)	0.83
No	1096.00						
Phases							
Not applicable	973.00	1.09 (1.03–1.16)	< 0.01	1.06 (0.98–1.14)	0.16	1.07 (0.96–1.19)	0.22
Phase 1	360.00						
Phase 2	1055.00						
Phase 3	1036.00						
Phase 4	731.00						
Enrollment							
≤ 50	671.00	0.76 (0.71–0.82)	< 0.01	0.77 (0.70–0.84)	< 0.01	0.80 (0.71–0.90)	< 0.01
> 50 and ≤ 100	725.00						
> 100 and ≤ 200	1096.00						
> 200 and ≤ 400	1308.00						
> 400	1646.00						
Funded by the NIH							
Yes	2007.00	0.44 (0.27–0.70)	< 0.01	0.47 (0.28–0.82)	0.01	0.41 (0.22–0.78)	0.01
No	894.00						
Funded by industry							
Yes	762.00	0.88 (0.69–1.14)	0.33	0.96 (0.72–1.28)	0.79	1.09 (0.74–1.60)	0.66
No	945.00						
Allocation							
Not applicable	700.00	0.92 (0.80–1.06)	0.27	0.92 (0.72–1.18)	0.52	0.91 (0.65–1.28)	0.59
Non-randomized	851.00						
Randomized	1003.00						
Intervention model							
Single	819.00	1.01 (0.91–1.13)	0.83	1.13 (0.95–1.35)	0.16	1.18 (0.93–1.49)	0.18
Sequential	744.00						
Parallel	1004.00						
Factorial	1065.00						
Crossover	365.00						
Masking							
None	941.00	1.09 (1.01–1.17)	0.02	1.00 (0.92–1.10)	0.93	0.98 (0.87–1.11)	0.75
Single	1037.00						
Double	819.00						
Triple	671.00						
Quadruple	1055.00						
Primary purpose							
Diagnostic							
Yes	638.00	1.78 (1.08–2.94)	0.04	1.35 (0.66–2.74)	0.41	1.73 (0.60–5.00)	0.31
No	950.00						
Health service research							
Yes	950.00	0.98 (0.56–1.70)	0.94	0.81 (0.39–1.70)	0.58	0.62 (0.20–1.89)	0.40
No	931.00						
Prevention							
Yes	822.00	1.08 (0.85–1.39)	0.53	1.01 (0.59–1.71)	0.98	0.94 (0.44–2.01)	0.88
No	1003.00						
Supportive care							
Yes	747.00	1.24 (0.84–1.83)	0.30	1.10 (0.60–2.02)	0.76	0.88 (0.38–2.04)	0.77
No	945.00						
Treatment							
Yes	1047.00	0.83 (0.68–1.01)	0.06	0.76 (0.46–1.24)	0.26	0.75 (0.37–1.52)	0.42
No	762.00						
Interventions							
Behavioral							
Yes	882.00	0.96 (0.68–1.37)	0.83	1.16 (0.78–1.72)	0.46	0.95 (0.58–1.55)	0.82
No	941.00						
Drug/biological							
Yes	731.00	1.57 (1.28–1.92)	< 0.01	1.48 (1.07–2.05)	0.02	1.50 (0.96–2.35)	0.07
No	1035.00						
Device							
Yes	1096.00	0.71 (0.58–0.88)	< 0.01	0.93 (0.70–1.24)	0.63	0.91 (0.60–1.40)	0.68
No	866.00						
Procedure							
Yes	914.00	0.98 (0.80–1.20)	0.83	1.07 (0.82–1.40)	0.60	0.75 (0.50–1.14)	0.17
No	931.00						
Location in China							
Yes	866.00	1.25 (0.92–1.71)	0.17	0.93 (0.64–1.34)	0.68	0.56 (0.28–1.10)	0.09
No	973.00						
Location in the United States							
Yes	1125.00	0.79 (0.63–0.99)	0.03	0.63 (0.46–0.87)	< 0.01	0.56 (0.35–0.90)	0.02
No	882.00						
Location in Europe							
Yes	1065.00	0.82 (0.68–0.98)	0.03	0.62 (0.48–0.81)	< 0.01	0.51 (0.35–0.76)	< 0.01
No	821.00						
Location in Canada							
Yes	931.00	1.09 (0.75–1.57)	0.66	0.69 (0.45–1.05)	0.08	0.54 (0.30–0.97)	0.04
No	945.00						

*NIH* National Institutes of Health, *HR* Hazard ratio, *CI* Confidence intervals

^a^indicates *P* values obtained from the analysis excluding estimated completion time

### Risk factors associated with successful trial completion (having results on the ClinicalTrials.gov website)

On the basis of multiple logistic regression analysis, enrollment (*P* = 0.04), being funded by the NIH (*P* = 0.02), being funded by industry (*P* < 0.01), drug/biological therapy (*P* = 0.03), and location in the United States (*P* < 0.01) were significantly associated with successful trial completion (Table 5). In particular, smaller enrollment numbers, being funded by the NIH or industry, involving drugs/biologics, and location in the United States were associated with a higher rate of successful trial completion. To investigate the predictive performance of the five mentioned significant variables, their AUROCs were calculated.

**Table 5:** Univariate and multivariate analyses of characteristics for successful trial completion (having results on ClinicalTrials.gov)

Characteristics	Rates	Simple logistic regression		Multiple logistic regression	
		**OR (95% CI)**	**P**	**OR (95% CI)**	**P**
Outcome measures including mortality					
Yes	6.25% (5/80)	0.64 (0.24–1.67)	0.36	1.25 (0.35–4.49)	0.73
No	9.49% (37/390)				
Gender					
Male	25.00% (1/4)	2.08 (0.35–12.28)	0.42	3.07 (0.42–22.46)	0.27
Female	6.67% (1/15)				
Both	8.87% (40/451)				
Age including children					
Yes	5.00% (2/40)	0.51 (0.12–2.21)	0.37	1.30 (0.22–7.63)	0.77
No	9.30% (40/430)				
Age including older adults					
Yes	8.89% (41/461)	0.78 (0.10–6.40)	0.82	0.96 (0.08–11.59)	0.97
No	11.11% (1/9)				
Phases					
Not applicable	5.71% (18/315)	1.30 (1.08–1.55)	< 0.01	1.05 (0.77–1.44)	0.74
Phase 1	0.00% (0/4)				
Phase 2	25.93% (7/27)				
Phase 3	16.33% (8/49)				
Phase 4	12.00% (9/75)				
Enrollment					
≤ 50	12.90% (16/124)	0.89 (0.69–1.15)	0.39	0.71 (0.51–0.99)	0.04
> 50 and ≤ 100	7.14% (9/126)				
> 100 and ≤ 200	6.60% (7/106)				
> 200 and ≤ 400	7.35% (5/68)				
> 400	10.87% (5/46)				
Funded by the NIH					
Yes	33.33% (6/18)	5.78 (2.05–16.31)	< 0.01	7.55 (1.48–38.56)	0.02
No	7.96% (36/452)				
Funded by industry					
Yes	23.61% (17/72)	4.61 (2.34–9.09)	< 0.01	6.32 (2.43–16.44)	< 0.01
No	6.28% (25/398)				
Allocation					
Not applicable	1.92% (1/52)	1.53 (0.82–2.85	0.18	1.28 (0.42–3.92)	0.66
Non-randomized	13.89% (5/36)				
Randomized	9.42% (36/382)				
Intervention model					
Single	3.85% (3/78)	1.30 (0.89–1.91)	0.18	1.54 (0.76–3.10)	0.23
Sequential	0.00% (0/3)				
Parallel	10.19% (37/363)				
Factorial	11.11% (1/9)				
Crossover	5.88% (1/17)				
Masking					
None	9.68% (18/186)	1.24 (0.99–1.56)	0.06	0.83 (0.59–1.17)	0.28
Single	4.80% (6/125)				
Double	3.61% (3/83)				
Triple	21.43% (6/28)				
Quadruple	18.75% (9/48)				
Primary purpose					
Diagnostic					
Yes	6.25% (1/16)	0.67 (0.09–5.21)	0.70	0.41 (0.01–24.96)	0.67
No	9.03% (41/454)				
Health service research					
Yes	0.00% (0/13)	0.01 (0.00–99.99)	0.98	0.01 (0.00–99.99)	0.98
No	9.19% (42/457)				
Prevention					
Yes	13.51% (10/74)	1.78 (0.83–3.79)	0.14	1.20 (0.10–14.56)	0.89
No	8.08% (32/396)				
Supportive care					
Yes	0.00% (0/27)	0.01 (0.00–99.99)	0.98	0.01 (0.00–99.99)	0.98
No	9.48% (42/443)				
Treatment					
Yes	9.38% (30/320)	1.19 (0.59–2.39)	0.63	0.70 (0.07–7.40)	0.77
No	8.00% (12/150)				
Interventions					
Behavioral					
Yes	2.94% (1/34)	0.29 (0.04–2.19)	0.23	0.18 (0.01–2.52)	0.20
No	9.40% (41/436)				
Drug/biological					
Yes	17.61% (25/142)	3.91 (2.04–7.50)	< 0.01	4.47 (1.19–16.83)	0.03
No	5.18% (17/328)				
Device					
Yes	12.17% (14/115)	1.62 (0.82–3.19)	0.16	2.53 (0.77–8.29)	0.12
No	7.89% (28/355)				
Procedure					
Yes	4.55% (6/132)	0.40 (0.16–0.97)	0.04	0.99 (0.34–2.90)	0.99
No	10.65% (36/338)				
Location in China					
Yes	4.55% (2/44)	0.46 (0.11–1.97)	0.30	1.91 (0.26–13.86)	0.52
No	9.39% (40/426)				
Location in the United States					
Yes	29.00% (29/100)	11.22 (5.56–22.63)	< 0.01	9.43 (2.41–36.95)	< 0.01
No	3.51% (13/370)				
Location in Europe					
Yes	3.06% (6/196)	0.21 (0.09–0.51)	< 0.01	0.89 (0.20–4.00)	0.88
No	13.14% (36/274)				
Location in Canada					
Yes	6.45% (2/31)	0.69 (0.16–2.99)	0.62	1.96 (0.25–15.67)	0.53
No	9.11% (40/439)				

*OR* Odds rates, *NIH* National Institutes of Health, *CI* Confidence intervals

**Fig. 5:**
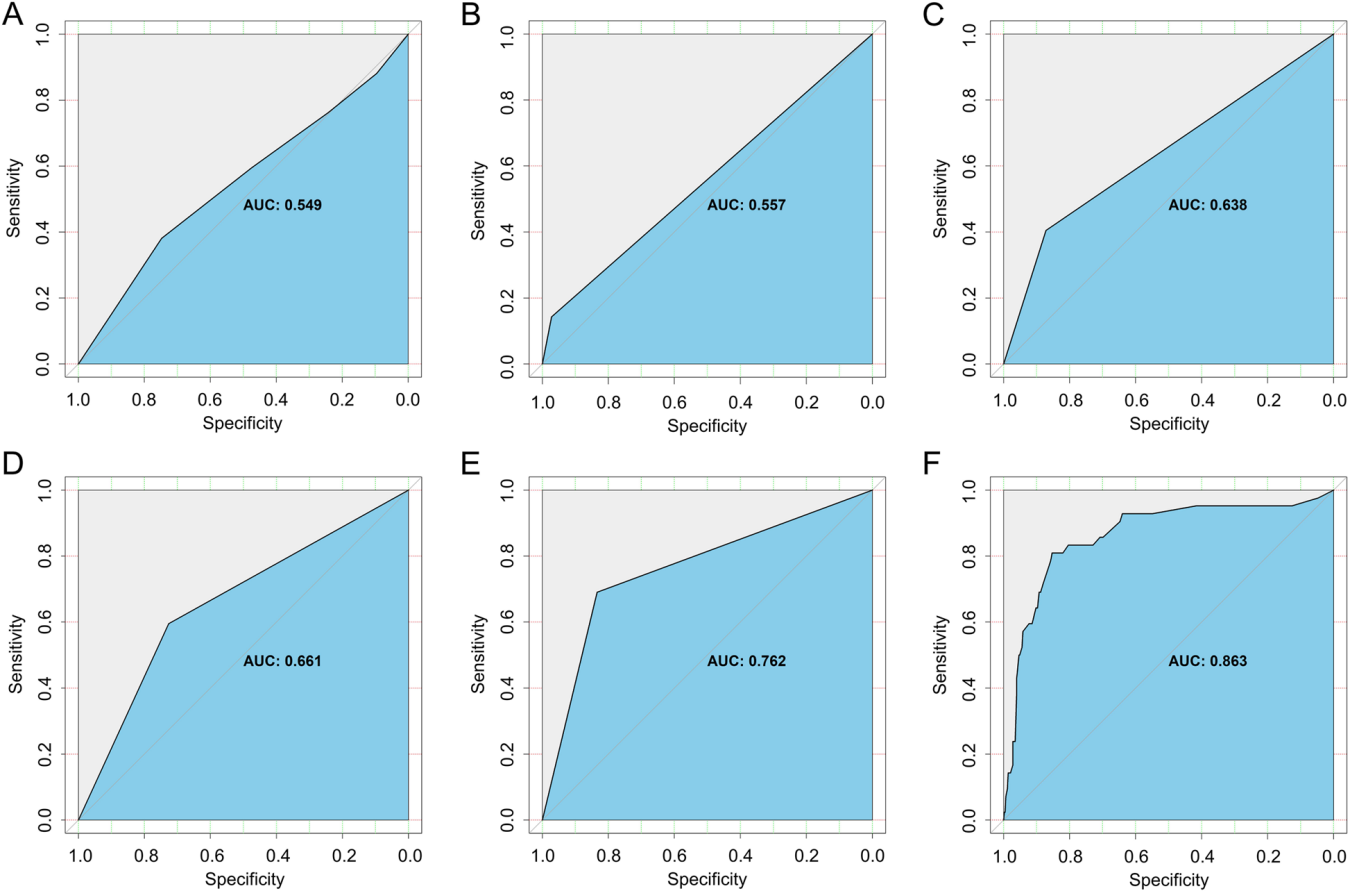
The area under the receiver operating characteristic (AUROC) for the five significant variables associated with successful trial completion (having results on the ClinicalTrial.gov website). **a** Enrollment alone; **b** Funded by the National Institutes of Health alone; **c** Funded by industry alone; **d** Interventions involving drug/biological agents alone; **e** Location in the United States alone; **f** Combination of the five variables

The AUROCs for enrollment alone (Fig. 5A), being funded by the NIH alone (Fig. 5B), being funded by industry alone (Fig. 5C), drug/biological therapy alone (Fig. 5D), and location in the United States alone (Fig. 5E) were 0.55, 0.56, 0.64, 0.66, and 0.76, respectively. On combining the five variables, the total AUROC rose to 0.86 (Fig. 5F), indicating favorable prediction performance of these variables.

## Discussion

Our study presented and summarized the data from clinical trials related to hip fractures and found that 44.89% (211/470) of the included trials had already completed recruitment; the overall median completion time was approximately 2.6 years. This number was relatively higher than that reported in other studies. In 2018, DePasse et al. [6] reported the completion rate of spinal cord injury trials registered at ClinicalTrials.gov to be 39.9%. After analyzing 97 clinical trials related to the use of artificial intelligence in the diagnosis of cancers in 2020, Dong et al. [9] found the trial completion rate to be 15.4%. In 2012, Califf et al. [10] analyzed the data from 96,346 clinical studies registered between 2007 and 2010; they found the completion rate to be 29.9%. Heterogeneity of the completion rates may be explained by wide variability in the diseases, advancement of technologies, and registered dates.

However, only 8.94% (42/470) of clinical trials in our study had published results on the ClinicalTrials.gov website; this indicates that it is necessary to improve transparency of trial data reporting, irrespective of positive or negative results. In this context, studies have demonstrated that researchers are more likely to report positive results [11]. Notably, improved prospective registration and consistent reporting of results on the ClinicalTrials.gov website will help the public find reliable information and avoid potential bias [12]. Our study found that smaller enrollment numbers, being funded by the NIH or industry, use of drugs/biologics, and location in the United States were associated with a higher rate of successful trial completion on the ClinicalTrial.gov website. Smaller enrollment numbers appeared to be associated with a higher completion rate and shorter completion time; this may have increased the likelihood of presentation of results. Clinical trials being funded by the NIH or industry may need to abide by strict laws, policies, and scientific norms/practices, as these trials are relatively large and of a multinational design. Various incentives and rules may influence the trial components [13].

In clinical trials testing drugs/biologics for the prevention and treatment of hip fractures, the top three agents were bupivacaine (a local anesthetic, 25 clinical trials), ropivacaine (a local anesthetic, 23 clinical trials) and tranexamic acid (a hemostatic, 21 clinical trials). Regarding the pharmacological action of the agents appearing in 3 or more clinical trials, 4 of the 14 agents were anti-osteoporotic, 3 were local anesthetics, and 2 were pain killers. We could not therefore conclude whether the majority of agents focused on anti-osteoporosis, pain alleviation, or hemostasis.

The findings of this study indicated that trials including children as participants (*P* = 0.03) and those funded by the NIH (*P* < 0.01) had a significantly higher likelihood of complete recruitment. In particular, clinical trials involving children were twice as likely to complete recruitment compared to those not involving children. Clinical trials funded by the NIH were 8.71 times more likely to have completed recruitment status as compared to those not funded by the NIH. However, clinical trials that were funded by the NIH also had longer completion time. The results therefore indicated that clinical trials that were funded by the NIH may have been relatively large and had strict requirements or processes. In addition, participants may have had more trust in these studies, and were more willing to participate. Therefore, clinical trials funded by the NIH needed more time to complete and had higher complete recruitment rates. Regarding subgroup analysis, outcome measures including mortality were associated with a higher recruitment rate (21.25% vs. 13.85%) and a lower suspension rate (5.00% vs. 10.51%); this indicated that clinical trials evaluating patient mortality were less likely to be stopped early, as adequate time was needed to measure patient survival outcomes. The 1-year mortality in some studies was found to be 26%-33% [14, 15], and the median overall survival of patients with hip fractures was 886 days [15]. This may also indicate that selection of mortality as an outcome was more likely to be associated with successful completion, although these trials needed more time to complete. A later phase of clinical trials tended to be associated with a lower completion rate; this could be explained by stricter rules and regulations in later phases. Enrollment of more than 200 and less than 400 participants was associated with the highest completion rate; this indicated that appropriate enrollment facilitated completion. This study also found that higher enrollment, being funded by the NIH, and location in the United States or Europe tended to prolong completion time, while the use of drugs/biologics was associated with shorter completion time. The above results suggested that an appropriate number of participants/sponsors and tested drugs facilitated completion. However, it is necessary to achieve a balance, as trials funded by the NIH and those performed in the United States may have been relatively large, requiring higher enrollment and completion time. In this context, industry-funded trials for rare diseases are less vulnerable to discontinuation than healthcare center-funded trials [16].

### Limitations

The present study has certain limitations. Firstly, ClinicalTrials.gov did not include all the clinical trials worldwide; this may limit the generalization of our results to other aspects and areas. Nonetheless, ClinicalTrials.gov has already registered over 80% of all clinical trials on the World Health Organization platform [17]. Secondly, missing and incomplete data may have led to potential bias [18]; however, as the present study excluded clinical studies with missing data, bias may have been largely avoided. Lastly, dynamic changes of data always exist over time, and this study had a cross-sectional design. However, our study analyzed all data from clinical trials related to hip fractures over the past 20 years; it therefore reflected the trend of developments and advancements among patients with hip fractures. Nevertheless, future investigations will be needed.

## Conclusions

Although a considerable number of clinical trials related to hip fractures have been completed, the results of only a small fraction have been presented on the ClinicalTrials.gov website. The commonly investigated drugs focus on anesthesia, pain relief, and hemostasis. Several independent risk factors have been identified to affect the recruitment status and completion time; these may guide the design of clinical trials related to hip fractures.

## Supplementary Information

**Additional file 1.**

**Additional file 2.**

**Additional file 3.**

**Additional file 4.**

## Data Availability

All data were publicly available at https://clinicaltrials.gov/.

## Abbreviations

AUROC: Area under the receiver operating characteristics curve
NIH: National Institutes of Health
CI: Confidence intervals
HR: Hazard ratio
OR: Odds ratio
