# Supplementary material for: Characteristics of clinical trials related to hip fractures and factors associated with completion
Source: BMC Musculoskelet Disord. 2022 Aug 16;23:781. doi: 10.1186/s12891-022-05714-x (PMC9380385; doi:10.1186/s12891-022-05714-x)

Figure S2. Relative proportion of the number of new clinical trials against the time (years). The x-axis represents the years and the y-axis represents the relative abundance. Red indicates China, green indicates the United States, blue indicates Europe, cyan indicates Canada, and purple indicates others.

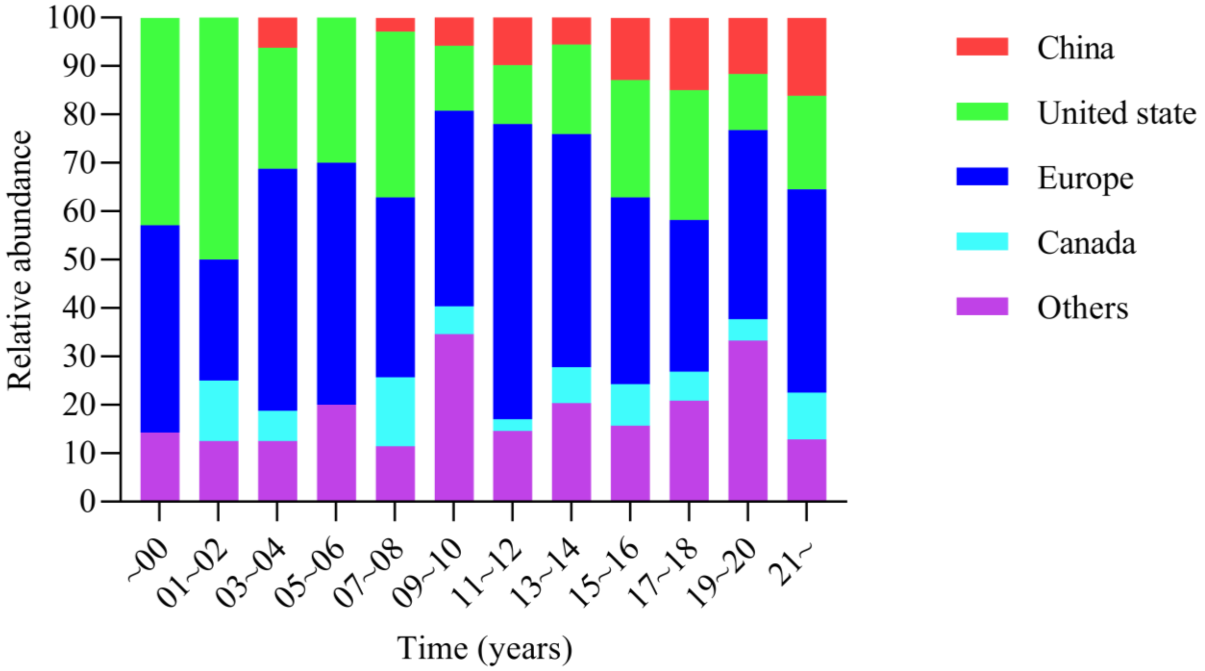

Supplement: Supplementary file 2 — Additional file 2. [file 12891_2022_5714_MOESM2_ESM.pdf]
