# Supplementary material for: Characteristics of clinical trials related to hip fractures and factors associated with completion
Source: BMC Musculoskelet Disord. 2022 Aug 16;23:781. doi: 10.1186/s12891-022-05714-x (PMC9380385; doi:10.1186/s12891-022-05714-x)
Supplement: Supplementary file 3 — Additional file 3. [file 12891_2022_5714_MOESM3_ESM.docx]

| **Table S1** Univariate and multivariate analyses of characteristics and recruitment status in clinical trials related to hip fractures. | | | | | |
| --- | --- | --- | --- | --- | --- |
| **Characteristics** | **Completion rates** | **Simple logistic regression** | | **Multiple logistic regression** | |
|  |  | **OR (95% CI)** | **P** | **OR (95% CI)** | **P** |
| Outcome measures including mortality |  |  |  |  |  |
| Yes | 45.00% (36/80) | 0.62 (0.40-0.96) | 0.03 | 0.61 (0.37-0.98) | 0.04 |
| No | 44.87% (175/390) |  |  |  |  |
| Gender |  |  |  |  |  |
| Male | 75.00% (3/4) | 1.01 (0.44-2.32) | 0.98 | 1.19 (0.50-2.88) | 0.69 |
| Female | 53.33% (8/15) |  |  |  |  |
| Both | 44.35% (200/451) |  |  |  |  |
| Age including children |  |  |  |  |  |
| Yes | 57.50% (23/40) | 1.50 (0.82-2.73) | 0.18 | 1.41 (0.75-2.66) | 0.29 |
| No | 43.72% (188/430) |  |  |  |  |
| Age including older adults |  |  |  |  |  |
| Yes | 45.34% (209/461) | 0.82 (0.24-2.74) | 0.74 | 1.02 (0.28-3.73) | 0.97 |
| No | 22.22% (2/9) |  |  |  |  |
| Phases |  |  |  |  |  |
| Not applicable | 40.63% (128/315) | 1.13 (1.02-1.25) | 0.02 | 1.20 (1.05-1.38) | 0.01 |
| Phase 1 | 50.00% (2/4) |  |  |  |  |
| Phase 2 | 70.37% (19/27) |  |  |  |  |
| Phase 3 | 61.22% (30/49) |  |  |  |  |
| Phase 4 | 42.67% (32/75) |  |  |  |  |
| Enrollment |  |  |  |  |  |
| ≤50 | 43.55% (54/124) | 0.82 (0.72-0.93) | <0.01 | 0.84 (0.73-0.98) | 0.02 |
| >50 and ≤100 | 42.86% (54/126) |  |  |  |  |
| >100 and ≤200 | 47.17% (50/106) |  |  |  |  |
| >200 and ≤400 | 54.41% (37/68) |  |  |  |  |
| >400 | 34.78% (16/46) |  |  |  |  |
| Funded by NIH |  |  |  |  |  |
| Yes | 83.33% (15/18) | 1.24 (0.52-2.96) | 0.62 | 2.14 (0.77-5.94) | 0.15 |
| No | 43.36% (196/452) |  |  |  |  |
| Funded by industry |  |  |  |  |  |
| Yes | 50.00% (36/72) | 0.65 (0.41-1.02) | 0.06 | 0.71 (0.43-1.16) | 0.17 |
| No | 43.97% (175/398) |  |  |  |  |
| Allocation |  |  |  |  |  |
| Not applicable | 32.69% (17/52) | 1.04 (0.81-1.34) | 0.77 | 1.52 (1.01-2.29) | 0.05 |
| Non-randomized | 50.00% (18/36) |  |  |  |  |
| Randomized | 46.07% (176/382) |  |  |  |  |
| Intervention model |  |  |  |  |  |
| Single | 42.31% (33/78) | 0.91 (0.75-1.10) | 0.33 | 0.81 (0.61-1.07) | 0.14 |
| Sequential | 0.00% (0/3) |  |  |  |  |
| Parallel | 45.18% (164/363) |  |  |  |  |
| Factorial | 66.67% (6/9) |  |  |  |  |
| Crossover | 47.06% (8/17) |  |  |  |  |
| Masking |  |  |  |  |  |
| None | 44.09% (82/186) | 0.95 (0.83-1.07) | 0.39 | 0.87 (0.74-1.02) | 0.09 |
| Single | 45.60% (57/125) |  |  |  |  |
| Double | 45.78% (38/83) |  |  |  |  |
| Triple | 42.86% (12/28) |  |  |  |  |
| Quadruple | 45.83% (22/48) |  |  |  |  |
| Primary purpose |  |  |  |  |  |
| Diagnostic |  |  |  |  |  |
| Yes | 25.00% (4/16) | 1.83 (0.72-4.61) | 0.20 | 3.58 (1.03-12.46) | 0.04 |
| No | 45.59% (207/454) |  |  |  |  |
| Health service research |  |  |  |  |  |
| Yes | 46.15% (6/13) | 0.84 (0.31-2.29) | 0.74 | 1.53 (0.42-5.59) | 0.52 |
| No | 44.86% (205/457) |  |  |  |  |
| Prevention |  |  |  |  |  |
| Yes | 40.54% (30/74) | 1.20 (0.76-1.90) | 0.43 | 2.10 (0.81-5.47) | 0.13 |
| No | 45.71% (181/396) |  |  |  |  |
| Supportive care |  |  |  |  |  |
| Yes | 44.44% (12/27) | 0.58 (0.29-1.17) | 0.13 | 0.86 (0.29-2.54) | 0.79 |
| No | 44.92% (199/443) |  |  |  |  |
| Treatment |  |  |  |  |  |
| Yes | 47.19% (151/320) | 1.08 (0.76-1.54) | 0.67 | 1.57 (0.67-3.71) | 0.30 |
| No | 40.00% (60/150) |  |  |  |  |
| Interventions |  |  |  |  |  |
| Behavioral |  |  |  |  |  |
| Yes | 61.76% (21/34) | 1.26 (0.66-2.38) | 0.49 | 1.46 (0.70-3.04) | 0.32 |
| No | 43.58% (190/436) |  |  |  |  |
| Drug/biological |  |  |  |  |  |
| Yes | 50.00% (71/142) | 1.16 (0.81-1.67) | 0.41 | 1.04 (0.60-1.79) | 0.89 |
| No | 42.68% (140/328) |  |  |  |  |
| Device |  |  |  |  |  |
| Yes | 38.26% (44/115) | 0.86 (0.58-1.25) | 0.42 | 1.05 (0.65-1.69) | 0.84 |
| No | 47.04% (167/355) |  |  |  |  |
| Procedure |  |  |  |  |  |
| Yes | 40.91% (54/132) | 1.25 (0.86-1.81) | 0.24 | 1.28 (0.83-1.97) | 0.27 |
| No | 46.45% (157/338) |  |  |  |  |
| Location in China |  |  |  |  |  |
| Yes | 27.27% (12/44) | 1.33 (0.75-2.36) | 0.33 | 0.83 (0.42-1.64) | 0.59 |
| No | 46.71% (199/426) |  |  |  |  |
| Location in the United States |  |  |  |  |  |
| Yes | 49.00% (49/100) | 0.67 (0.45-1.00) | 0.05 | 0.40 (0.23-0.70) | <0.01 |
| No | 43.78% (162/370) |  |  |  |  |
| Location in Europe |  |  |  |  |  |
| Yes | 47.45% (93/196) | 0.95 (0.68-1.32) | 0.75 | 0.69 (0.44-1.10) | 0.12 |
| No | 43.07% (118/274) |  |  |  |  |
| Location in Canada |  |  |  |  |  |
| Yes | 45.16% (14/31) | 0.90 (0.46-1.75 | 0.76 | 0.58 (0.27-1.25) | 0.16 |
| No | 44.87% (197/439) |  |  |  |  |
| NIH, national institutes of health; OR, Odds ratio; CI, confident interval. | | | | | |
