# Supplementary material for: Characteristics of clinical trials related to hip fractures and factors associated with completion
Source: BMC Musculoskelet Disord. 2022 Aug 16;23:781. doi: 10.1186/s12891-022-05714-x (PMC9380385; doi:10.1186/s12891-022-05714-x)
Supplement: Supplementary file 4 — Additional file 4. [file 12891_2022_5714_MOESM4_ESM.docx]

| **Table S2** Distribution of recruitment status among the six corresponding significant variables. | | | | | | | | | |
| --- | --- | --- | --- | --- | --- | --- | --- | --- | --- |
| **Characteristics** | **Total**  **(n=470)** | **Recruitment status** | | | | | | | P |
|  |  | **Not yet recruiting** | **Recruiting** | **Enrolling by invitation** | **Active, not recruiting** | **Suspended** | **Completed** | **Unknown** |  |
| Outcome measures including mortality |  |  |  |  |  |  |  |  |  |
| Yes | 80 | 8.75% (7/80) | 21.25% (17/80) | 2.50% (2/80) | 6.25% (5/80) | 5.00% (4/80) | 45.00% (36/80) | 11.25% (9/80) | 0.02* |
| No | 390 | 6.41% (25/390) | 13.85% (54/390) | 1.28% (5/390) | 3.33% (13/390) | 10.51% (41/390) | 44.87% (175/390) | 19.74% (77/390) |  |
| Phases |  |  |  |  |  |  |  |  |  |
| Not applicable | 315 | 8.89% (28/315) | 17.78% (56/315) | 1.59% (5/315) | 5.08% (16/315) | 6.98% (22/315) | 40.63% (128/315) | 19.05% (60/315) | 0.01 |
| Phase 1 | 4 | 0.00% (0/4) | 25.00% (1/4) | 0.00% (0/4) | 0.00% (0/4) | 0.00% (0/4) | 50.00% (2/4) | 25.00% (1/4) |  |
| Phase 2 | 27 | 3.70% (1/27) | 7.41% (2/27) | 0.00% (0/27) | 3.70% (1/27) | 11.11% (3/27) | 70.37% (19/27) | 3.70% (1/27) |  |
| Phase 3 | 49 | 2.04% (1/49) | 8.16% (4/49) | 2.04% (1/49) | 0.00% (0/49) | 18.37% (9/49) | 61.22% (30/49) | 8.16% (4/49) |  |
| Phase 4 | 75 | 2.67% (2/75) | 10.67% (8/75) | 1.33% (1/75) | 1.33% (1/75) | 14.67% (11/75) | 42.67% (32/75) | 26.67% (20/75) |  |
| Enrollment |  |  |  |  |  |  |  |  |  |
| ≤50 | 124 | 3.23% (4/124) | 6.45% (8/124) | 2.42% (3/124) | 3.23% (4/124) | 22.58% (28/124) | 43.55% (54/124) | 18.55% (23/124) | <0.01 |
| >50 and ≤100 | 126 | 5.56% (7/126) | 15.87% (20/126) | 0.00% (0/126) | 3.17% (4/126) | 7.14% (9/126) | 42.86% (54/126) | 25.40% (32/126) |  |
| >100 and ≤200 | 106 | 9.43% (10/106) | 18.87% (20/106) | 0.00% (0/106) | 4.72% (5/106) | 2.83% (3/106) | 47.17% (50/106) | 16.98% (18/106) |  |
| >200 and ≤400 | 68 | 7.35% (5/68) | 17.65% (12/68) | 1.47% (1/68) | 0.00% (0/68) | 2.94% (2/68) | 54.41% (37/68) | 16.18% (11/68) |  |
| >400 | 46 | 13.04% (6/46) | 23.91% (11/46) | 6.52% (3/46) | 10.87% (5/46) | 6.52% (3/46) | 34.78% (16/46) | 4.35% (2/46) |  |
| Allocation |  |  |  |  |  |  |  |  |  |
| Not applicable | 52 | 11.54% (6/52) | 9.62% (5/52) | 3.85% (2/52) | 1.92% (1/52) | 17.31% (9/52) | 32.69% (17/52) | 23.08% (12/52) | 0.20 |
| Non-randomized | 36 | 2.78% (1/36) | 13.89% (5/36) | 2.78% (1/36) | 8.33% (3/36) | 8.33% (3/36) | 50.00% (18/36) | 13.89% (5/36) |  |
| Randomized | 382 | 6.54% (25/382) | 15.97% (61/382) | 1.05% (4/382) | 3.66% (14/382) | 8.64% (33/382) | 46.07% (176/382) | 18.06% (69/382) |  |
| Diagnostic |  |  |  |  |  |  |  |  |  |
| Yes | 16 | 0.00% (0/16) | 12.50% (2/16) | 6.25% (1/16) | 0.00% (0/16) | 18.75% (3/16) | 25.00% (4/16) | 37.50% (6/16) | 0.10 |
| No | 454 | 7.05% (32/454) | 15.20% (69/454) | 1.32% (6/454) | 3.96% (18/454) | 9.25% (42/454) | 45.59% (207/454) | 17.62% (80/454) |  |
| Location in the United States |  |  |  |  |  |  |  |  |  |
| Yes | 100 | 6.00% (6/100) | 11.00% (11/100) | 4.00% (4/100) | 9.00% (9/100) | 14.00% (14/100) | 49.00% (49/100) | 7.00% (7/100) | <0.01 |
| No | 370 | 7.03% (26/370) | 16.22% (60/370) | 0.81% (3/370) | 2.43% (9/370) | 8.38% (31/370) | 43.78% (162/370) | 21.35% (79/370) |  |
| * indicates that the P value was from the Mantel-Haenszel Chi-square test. | | | | | | | | | |
